# Neuroenhancement as Instrumental Drug Use: Putting the Debate in a Different Frame

**DOI:** 10.3389/fpsyt.2020.567497

**Published:** 2020-09-04

**Authors:** Stephan Schleim

**Affiliations:** Theory and History of Psychology, Heymans Institute for Psychological Research, Faculty of Behavioral and Social Sciences, University of Groningen, Groningen, Netherlands

**Keywords:** enhancement, addiction medicine, drug policy, stimulant drugs, drug instrumentalization

## Abstract

The use of performance-enhancing drugs to study or work better is often called “cognitive enhancement” or “neuroenhancement” and sparked a debate between scholars from many disciplines. I argue that such behavior can better be subsumed under the more general category of “instrumental drug use”. This broader perspective allows understanding neuroenhancement better from the perspective of addiction medicine and public health and supports a more consistent drug policy. I also summarize the most important systematic reviews and individual surveys of nonmedical substance use to study or work better. Different definitions and methodologies limit the comparability of these studies. The unified approach of drug instrumentalization would partially solve such problems. Finally, prevalence studies from the 1960s to 1980s as well as anecdotal evidence since the late 19th century show that instrumental drug use is and has been for a long time a common phenomenon. It should thus also be investigated and treated accordingly.

## Introduction

Neuroenhancement received a lot of attention by ethicists, legal scholars, pharmacologists, and researchers from further disciplines since the beginning of the 21^st^ century (1, 2).^1^ According to the analysis of O’Connor, Rees, and Joffe, enhancement of the brain was also the most frequent neuroscience-related topic in general news media (3). It actually received so much attention that Lucke and colleagues spoke of a “neuroenhancement bubble” (4). The substances discussed most intensively are the stimulant drugs amphetamine, methylphenidate, and its analogs, also known as treatments for attention and impulsivity disorders, as well as the wakefulness promoter modafinil (5–7). Presently, questions like whether the use of performance enhancing drugs at universities should be seen as cheating (8) or psychiatrists’ attitudes towards prescribing such substances in academic settings (9) are being discussed and investigated. The ethical and regulatory challenges of pharmacological enhancement were also addressed in this journal very recently (10).

The topic gained popularity with the rise of “neuroethics”, a new discipline specifically intended to cover ethical, legal, and social challenges related to the neurosciences (11). Along with some academic scholars, news media soon reported that neuroenhancement is a common and increasing phenomenon, in some cases literally “as common as coffee” (12). But a colleague and I showed earlier that such claims are often exaggerated (13–16). Describing neuroenhancement as new and increasing lent and still lends much of the urgency to this discussion and related research. We reported evidence, though, that the phenomenon, particularly the use of stimulant drugs in academic settings, exists at least since the 1930s (14, 15).

That people use drugs to change their psychological state in order to study or work better has been associated with many terms before: cognitive, affective, or neuroenhancement, brain doping, smart drugs, non-medical drug use, recreational drug use, substance abuse, and probably many more. Publications in neuroethics particularly used the term “enhancement” and discussed it with respect to ethical issues such as freedom of choice, individual and social performance improvement, coercion, and fairness (1, 2). In this paper, I want to put it in a different perspective: that of *instrumental drug use*. Hitherto, the discussion of neuroenhancement in neuroethics on the one hand and research on addiction, substance abuse, drug diversion (17) and diffusion (18) on the other have been proceeding mostly separate from each other. For example, the previously mentioned seminal and influential publications don’t even address the topic of addiction (1, 2). If we frame the phenomenon as instrumental drug use instead, we can easily integrate that which should never have been separate in the first place.

## Instrumental Drug Use

It is a matter of fact: Many people are and have been using substances to achieve different kinds of aims. This is what characterizes such use as *instrumental*: People are doing it for a certain purpose; the substance thus becomes an instrument to get what people want to get. Note that this also introduces a notion of rationality, if people are justifiably convinced that doing something increases the likelihood of reaching a specific goal, and are then doing it. Substance use can imply irrational aspects, too; for example, when consumers are taking a substance to achieve a certain goal but at the same time damage themselves or others. This would conflict with the aim of being healthy and maintaining good relations.^2^ People could also fail to stop using substances, even knowing that they harm themselves or others or that their use is getting out of control. This puts us into the domain of addiction, which has traditionally been discussed as an example for limiting human rationality and freedom. What I want to show here is that—at least within certain boundaries—substance use can be instrumental and thus rational. And substances affecting the nervous system on which our psychological functioning relies are in many instances called “drugs”. In that sense, drugs can be understood as instruments, which has particularly been elaborated theoretically as well as practically by Christian P. Müller (19, 20).

Müller argues that instrumental drug use has ancient roots in human and actually also non-human history and that it can have an adaptive value in evolution. Thus, animals being able to use drugs to increase their chances of survival and procreation would have evolutionary benefits. Specifically and for humans in the present society, he distinguished nine different goals of drug instrumentalization: (1) improving social interaction; (2) facilitating sexual behavior; (3) improving cognitive performance or counteracting fatigue; (4) facilitating recovery or coping with stress; (5) self-medication for psychiatric disorders and mental problems; (6) sensory curiosity (*e.g.* “expanding” one’s perceptions); (7) experiencing euphoria, hedonia, or a “high”; (8) improving physical appearance or attractiveness; and (9) facilitating spiritual or religious activities (19).

Note that just by distinguishing these nine goals one does not automatically approve of that behavior. In the debate, “enhancement” or “brain doping” might be positively or negatively biased concepts, respectively. In contrast to that, “instrumental drug use” seems a more neutral alternative, an alternative that provides us with a general analytical tool to make sense of people’s drug consumption. In the context of the neuroenhancement debate, particularly Müller’s first (social interaction), third (cognitive performance), and fourth (coping with stress) goal would be salient, as these are arguably relevant domains in present-day study and work environments. To demonstrate the usefulness of this analytical category, I shall discuss studies about the prevalence of instrumental drug use, from the past as well as the present, and then try to unify the different perspectives in the conclusion.

## Prevalence Studies

Dozens of studies addressed the prevalence of instrumental drug use in study or work contexts. Already in 2011, Smith and Farah reviewed evidence from 28 individual articles investigating students’ “nonmedical stimulant use” published between 2000 and 2009 (21). It is important to understand that methodologies varied widely: Most studies were based on self-reports from non-representative samples sized between N = 50 and N = 54,079 participants. The kinds of drugs included in the surveys differed (*e.g.* only prescription stimulants like methylphenidate or amphetamine or also illicit drugs like cocaine) as did the time spans (last month, last year, or lifetime use). Unsurprisingly, the outcomes then varied widely between 1.7 and 55%. The latter figure comes from a study investigating only fraternity members at a single location, a group that is notorious for its above-average drug use (22). Finally, not all studies asked for the motives of the nonmedical drug use and where they were surveyed answers also indicated recreational use like experiencing a “high” or partying better.

A very recent systematic review of nonmedical prescription stimulant use found already 111 studies meeting the inclusion criteria (23). But just like Smith and Farah before, Faraone and colleagues found much variance between the publications in terms of definitions used to investigate nonmedical use, methodologies, and samples, which made a formalized meta-analysis impossible. Similar to the earlier review, the prevalence of self-reported use varied between 2.1 and 58.7% and almost exclusively referred to student populations. The only population-based estimate, the US National Survey on Drug Use and Health 2015–2016, found that 2.1% adults had used stimulant drugs nonmedically at least once in the past year (24). Importantly, Faraone and colleagues discuss the reasons for which people had used the drugs in much detail. Academic motivations were mentioned most frequently. However, this may simply reflect that most participants were college students for whom this is particularly salient. The second most commonly mentioned reason was recreation (*e.g.* “getting high”, enhancing the effects of other drugs, help with socializing). Weight loss was cited less frequently as motivation. Summarizing all the data, males, 18–25 year-olds, whites, fraternity/sorority members, students with worse grades, people who had been binge drinking in the last month, had used marijuana or nonprescription stimulants (*e.g.* MDMA or methamphetamine) in the past year, had had adverse childhood experiences, and have not grown up with both biological parents were most likely to use prescription stimulants (23). The authors conclude that this use is a significant public health problem, but that it has not reached epidemic proportions like nonmedical opioid use in the United States.

One survey deserving individual attention is the recent cross-sectional study of pharmacological cognitive enhancement among non-ADHD individuals in 15 countries by Maier and colleagues (25). Their data are based on the Global Drug Survey of 2015 and 2017, jointly comprising responses of more than N = 100,000 subjects who filled out questionnaires anonymously on the internet in response to advertisements in offline and online media. Remarkably, the authors report an almost threefold increase of the 12-month prevalence of prescription stimulant, modafinil, and/or illegal stimulant use to increase performance when studying or working from, on average, 4.9 to 13.7% in the 15 countries (United States, Netherlands, United Kingdom, Canada, Belgium, Ireland, France, Australia, Hungary, Brazil, Austria, Switzerland, Germany, Portugal, and New Zealand; in the order of the prevalence in 2017, from highest to lowest). In some countries, the increase would have been *sixfold* (France, from 2.7 to 16.2%) or almost sixfold (Ireland, from 3.4 to 18.8%) in only *two years*.

However, the authors concede that the wording of the questions was changed between the two cohorts and that, while their annual surveys on drug use always run from November to January, the new module on performance enhancing use for the 2017 cohort had to be removed after only one month because it had made the survey too long (25). Therefore, the second cohort is almost two thirds smaller than the first. The authors conclude that the differences still reflect a real increase of stimulant use for performance enhancement. Yet, as I see it, there is the possibility that drug consumers were more motivated to respond quickly and to complete the long version of the survey than non-consumers. This shows that it is difficult to make reliable statements about an increase even by studies from the same research team if the procedure is not repeated *exactly* and not based on a representative sample. An interesting finding of the second cohort, though, is that cannabis, alcohol, and benzodiazepines, which have been added in the 2017 survey, were also mentioned as performance enhancement drugs by many, particularly to increase relaxation such that one could study or work better at a later time.

To my knowledge, the hitherto most reliable evidence for an increase is reported by McCabe and colleagues, who repeated a survey at a North American college six times in the period from 2003 to 2013 (17). The non-representative samples reported an increase from 5.4 to 9.3% for the past-year and from 8.1 to 12.7% for the lifetime nonmedical use of stimulant drugs, respectively. Unfortunately, the researchers did not publish their results on the frequency of consumption. Students might thus simply have tried to find out what the media hype described in the *Introduction* above is about and used the substances only a few times.^3^

I would now like to compare these systematic reviews and essential surveys from the 21^st^ century with studies published before 1990. To my knowledge, they have neither been addressed in the neuroethical debate nor in the studies summarized above. Smith and Blachly, for example, reported in 1966 that 92 (44%) of 208 medical students had used amphetamine at least once in their life (27). Of these consumers, 46% mentioned reduced fatigue, 11% reduced appetite, 10% improved alertness, 4% improved attention span, and also 4% increased motivation as benefits. Note that this is also instrumental drug use in the sense Müller described above (19, 20). In a much bigger sample of N = 7,170 New England college students, Wechsler and Rohman investigated, among other drugs, marijuana, stimulant, cocaine, tranquilizer, and hallucinogen consumption (28). The past-year prevalence reported in 1981 was 59.3, 16.2, 11.1, 9.6, and 7.8%, respectively. Of these consumers, 16% had used stimulants, 11% cocaine, and 10% tranquilizers to stay awake or study better, which we would nowadays often call “neuroenhancement”.

The most relevant study I found from this period is by McAuliffe and colleagues who reviewed prevalence data on nonmedical drug use by present and future health professionals in no less than 21 individual studies published between 1966 and 1980 (29). Besides recreational use and self-treatment, they also defined instrumental use “to stay awake, to fall asleep, or to perform better on tests or in sports” as nonmedical use. The lifetime prevalence of amphetamine consumption, which was mostly instrumental, ranged between 11 and 54% in these samples. The authors discuss that the instrumental amphetamine use would be declining since the mid-1960s, while the recreational use of other drugs would be increasing. The same authors published a survey of N = 337 physicians, N = 312 pharmacists, N = 381 medical, and N = 278 pharmacy students two years after their review (30). For 19 drugs, including several stimulants, sedatives, and opioids, the groups had a self-reported 33.3, 19.3, 43.6, and 41.1% past-year prevalence, respectively, for recreational, instrumental use, and self-treatment combined. The professionals reported an average of 43.7 consumptions in their life and the students between 64.6 and 65.7. The lifetime prevalence for instrumental use in particular, thus to stay awake, facilitate work, or sports, was 16% for the physicians and 17% for the medical students.

These figures suggest that nonmedical drug use in general and instrumental consumption to study or work better was common, perhaps even more common in the 1960s to 1980s than it is now in the early 21^st^ century. Anecdotal evidence for students consuming amphetamine to improve their academic performance can be traced back until the 1930s in the USA and the Netherlands (31–33). Instrumental stimulant drug use is likely even half a century older in Europe, where the German pharmacologist and military surgeon Theodor Aschenbrandt gave cocaine to Bavarian soldiers during a maneuver in 1883 and noted their increased capacity to endure hunger, strain, fatigue, and heavy burdens (34), which in turn inspired Sigmund Freud’s research on the drug (35).^4^

## Conclusion

A critical conclusion from the previous section is that the rationale of carrying out ever more surveys on consumption prevalence is questionable if they use such different definitions and methodologies that even after 60 years of research no clear picture emerges, not even on whether the substance use is increasing relevantly or not. A common view on drug instrumentalization could solve at least some of these issues. In any case, the evidence reviewed in this paper suggests that neuroenhancement, if one wants to use this term, was certainly not a new phenomenon in the early 2000s. That stimulant drugs and mostly amphetamine and methylphenidate, which are available since the 1930s to 1950s, still belong to the most frequently nonmedically used substances according to the studies summarized above supports that conclusion. Note that evidence that these drugs are actually enhancing the functioning of healthy people in real-life settings is scarce as the new meta-analysis by Roberts and colleagues demonstrates once more (7). Caviola and Faber argued earlier that the effects are probably not better than those of computer training, physical exercise, and healthy sleep (36). Additionally, the minor to modest effects of the drugs have to be balanced against their risks and side-effects.

Perhaps it was due to the enthusiasm of neuroethicists who emerged as a new profession in the early 2000s that such drug use was described as a new trend and framed first as “enhancement” and then as “neuroenhancement”. But does it make sense to discuss the old phenomenon under a new label? I proposed here to apply Müller’s general framework of instrumental drug use instead (19, 20). That a substantial amount of people use various kinds of substances to achieve certain goals is a fact. From a public health perspective, it appears to make no sense to treat the nine different aims distinguished by Müller differently, as the risks of the consumption of, say, amphetamine do not differ whether someone uses the drug to study longer or to party longer.^5^ We have seen that, by contrast, framing substance use to study or work better as “enhancement” carried the risk of not addressing the problem of addiction at all (1, 2). Interestingly, the new survey by Maier and colleagues also found that about 28% of the stimulant consumers taking the substances to increase their cognitive performance would like to use less, but fewer than 2% reported to actually seek help (25). This suggests that this could be an important field for addiction medicine.

Treating Müller’s nine goals differently on the legal or ethical level would require someone to take value judgments about which are morally better or worse aims. My considerations don’t amount to a radical legalization of all substances, but rather to a more consistent treatment of them, in accordance with persistent calls for a more science-based drug policy (37). Concurring with a critique voiced against Müller’s approach before by Wu, I would emphasize the importance to protect people from too much external pressure and coercion such that they are as autonomous as possible to choose for themselves (38, 39). The big difference between, for example, English- and German-speaking countries that came up in the survey by Maier and colleagues calls for follow-up studies investigating cultural, social, and economic factors explaining why so many more people take stimulant drugs in the former than in the latter. But also for this research the idea of drug instrumentalization would provide a viable and actually more fine-grained framework.

## Data Availability Statement

The original contributions presented in the study are included in the article/supplementary material; further inquiries can be directed to the corresponding author.

## Author Contributions

The author confirms being the sole contributor of this work and has approved it for publication.

## Funding

This publication has been supported by the “History of Neuroethics” grant by the Dutch Research Foundation (NWO), grant number 451-15-042.

## Conflict of Interest

The author declares that the research was conducted in the absence of any commercial or financial relationships that could be construed as a potential conflict of interest.

## Footnotes

1 These publications in the high impact journals *Nature Reviews Neuroscience* and *Nature* received 772 and 973 Google Scholar citations (as of August 18, 2020), respectively, and are still being cited nowadays.

2 It goes without saying that some consumers might simply not care about their own health or the wellbeing of others when trying to achieve their aims. Many athletes engaging in doping accept the possible—and in some cases even probable—health risks, perhaps even the risk of death, associated with performance-enhancing drug consumption. The philosophical discussion of which behaviors can be deemed rational and which not goes beyond the purview of this paper.

3 In personal correspondence, Sean McCabe referred to another study in which they addressed the frequency of consumption. There, indeed, 82.1% of past-year nonmedical users of prescription stimulants reported use on less than 10 occasions (26).

4 I am indebted to my colleague Jeremy Burman for drawing my attention to Freud's early research on cocaine.

5 Everything else being equal. Of course, it could be that common consumption patterns differ in that they are requiring higher doses or are combined with other risky behaviors for some purposes. But this rather seems to be a gradual than a principal difference.
